# Innovative Processing and Sterilization Techniques to Unlock the Potential of Silk Sericin for Biomedical Applications

**DOI:** 10.3390/gels11020114

**Published:** 2025-02-06

**Authors:** Anabela Veiga, Rosa Ana Ramírez-Jiménez, Víctor Santos-Rosales, Carlos A. García-González, Maria Rosa Aguilar, Luis Rojo, Ana L. Oliveira

**Affiliations:** 1 CBQF-Centro de Biotecnologia e Química Fina–Laboratório Associado, Universidade Católica Portuguesa, Escola Superior de Biotecnologia, Rua Diogo Botelho 1327, 4169-005 Porto, Portugal; s-anveiga@ucp.pt; 2 LEPABE-Laboratory for Process Engineering, Environment, Biotechnology & Energy, Department of Chemical Engineering, Faculty of Engineering, University of Porto, R. Dr. Roberto Frias, 4200-465 Porto, Portugal; 3 ALiCE-Associate Laboratory in Chemical Engineering, Faculty of Engineering, University of Porto, Rua Dr. Roberto Frias, 4200-465 Porto, Portugal; 4 Instituto de Ciencia y Tecnología de Polímeros ICTP-CSIC, C. Juan de la Cierva, 3, 28006 Madrid, Spain; 5 AerogelsLab, I+D Farma Group (GI-1645), Department of Pharmacology, Pharmacy and Pharmaceutical Technology, Faculty of Pharmacy, iMATUS and Health Research Institute of Santiago de Compostela (IDIS), Universidade de Santiago de Compostela, 15782 Santiago de Compostela, Spain; 6 Centro de Investigación Biomédica en Red de Bioingienería, Biomateriales y Biotecnología CIBER-BBN, Instituto de Salud Carlos III, Calle Monforte de Lemos S/N, 28029 Madrid, Spain

**Keywords:** silk sericin, biomedical applications, processing, sterilization, supercritical CO_2_ (scCO_2_)

## Abstract

Silk sericin (SS), a by-product of the textile industry, has gained significant attention for its biomedical potential due to its biocompatibility and regenerative potential. However, the literature lacks information on SS processing methods and the resulting physicochemical properties. This study represents the first step in protocol optimization and standardization. In the present work, different processing techniques were studied and compared on SS extracted from boiling water: evaporation, rotary evaporation, lyophilization, and dialysis, which presented a recovery yield of approximately 27–32%. The goal was to find the most promising process to concentrate extracted SS solutions, and to ensure that the SS structure was highly preserved. As a result, a new cryo-lyophilization methodology was proposed. The proposed method allows for the preservation of the amorphous structure, which offers significant advantages including complete dissolution in water and PBS, an increase in storage stability, and the possibility of scaling-up, making it highly suitable for industrial and biomedical applications. The second part of the work focused on addressing another challenge in SS processing: efficient and non-destructive sterilization. Supercritical CO_2_ (scCO_2_) has been gaining momentum in the last years for sterilizing sensitive biopolymers and biological materials due to its non-toxicity and mild processing conditions. Thus, scCO_2_ technology was validated as a mild technique for the terminal sterilization of SS. In this way, it was possible to engineer a sequential cryo-lyophilization/scCO_2_ sterilization process which was able to preserve the original properties of this natural silk protein. Overall, we have valorized SS into a sterile, off-the-shelf, bioactive, and water-soluble material, with the potential to be used in the biomedical, pharmaceutical, or cosmetic industries.

## 1. Introduction

Silk sericin (SS) is a natural polymer that can be recovered from the silk textile industry and has increasingly gained interest from the scientific and industrial community due to its bioactive properties [1]. At the industrial scale, silk is obtained from genetically stable cocoons from silkworms, with the most common species being the Bombyx mori. Like collagen and gelatin, SS is a glue-like protein which displays biocompatibility and biodegradability. SS maintains the structural integrity of the silk fibers and represents 20–30% of their total weight [2].

SS comprises 18 amino acids, predominantly serine, glycine, and aspartic acid, thus making it a water-soluble protein [3]. SS mainly adopts the form of an amorphous random spiral, but may also present the form of a β-sheet organized structure. The random spiral easily acquires β-sheet conformation as a consequence of moisture absorption and mechanical elongation, forming a denser, organized, crystalline, and less soluble structure [4].

Over the last few years, increasing literature has demonstrated that SS has promising properties for the field of tissue engineering, such as antioxidant behavior and regenerative potential [5,6,7,8]. For instance, SS-treated wounds exhibit faster healing and lower levels of inflammatory mediators [5]. Moreover, SS stimulates collagen production [6], has antiaging and antiwrinkle effects [7], modulates the hydroxyapatite biomineralization process, and promotes cell adhesion and proliferation of mammalian cells when used as an organic matrix or medium for cell growth [8]. Additionally, it has been demonstrated that SS concentrations from 0.05 to 1 wt. % can prevent oxidative stress and improve culture development [9], reduce the risks of BSA retroviral infection [10], and enhance cell viability [11,12]. Owing to this fact, SS has been used in a multitude of industrial applications, such as biomedical engineering and cosmetics, in commercially available products [13,14,15]. 

Despite these advantages, SS processing and sterilization pose significant challenges that limit its widespread application. Existing degumming processing methods, such as high temperature and high pressure, chemical extraction, and enzymatic approaches, often lead to partial degradation of SS, loss of bioactive properties, or inconsistent quality [16]. Likewise, sterilization techniques such as ethylene oxide (EtO), gamma radiation, electron beam, steam, hydrogen peroxide plasma, and UV radiation are unsuitable for protein-based materials like SS, as they often alter molecular integrity, introduce toxic residues, or require extreme conditions [17,18]. Thus, there is an urgent need for innovative, scalable methods that preserve the intrinsic properties of SS while meeting industrial demands [13].

As for processing, one method of SS extraction involves boiling in water under ambient or increased pressure. Although resulting in lower yields, this has the advantage of not requiring any chemicals or introducing impurities, and is the simplest method [19,20,21,22]. Furthermore, SS can be used directly after extraction without any further purification steps [17].

To regenerate SS into 3D structures such as scaffolds and hydrogels, it is usually concentrated after extraction. As the SS concentration increases, its rate of gelation accelerates, and its hydrogel mechanical properties increase significantly [23]. Several techniques to increase concentration, including rotary evaporation [24], lyophilization [25], dialysis [26], and evaporation [27] have been reported. Evaporation relies on temperature to conduct concentration. Heating at high temperatures can cause some changes in the SS molecule [28]. However, major protein degradation peaks are only detectable when heated around 216 °C [29]. Although evaporation on a heating plate is challenging to control, this technique is simple and allows concentrations up to 14 wt. % to be obtained [27]. Lyophilization, where SS is dried and further regenerated to improve control over its concentration, has also been used in different works [30,31]. However, this process has been reported to induce β-sheet conformation which causes water insolubility [25,32]. Rotary evaporation has also been used to concentrate SS. However, the studies do not usually report all the relevant experimental parameters used (temperature, rotation, pressure, and final concentration), while temperatures between 85–60 °C and “low pressures” are often indicated [23,24,33]. Similarly, only limited information is available about dialysis, a time-consuming process that can be carried out using a PEG solution [26,34]. Although these techniques have been reported in the development of SS-based materials, there is no consensus on the best available method. Additionally, published studies lack information on the advantages, challenges, and most suitable final applications.

Concerning the sterilization phase, which is essential for both *in vitro* and *in vivo* testing, supercritical CO_2_ (scCO_2_) is gaining increasing attention as a sterilization agent able to inactivate vegetative forms of a wide range of Gram-positive and Gram-negative bacteria [35]. The mild operating conditions, low content of additives required, and excellent scCO_2_ permeability are clear advantages for the sterilization of biomaterials for biomedical applications [36]. The sterilization capability can be enhanced by incorporating small quantities of additives such as hydrogen peroxidase (H_2_O_2_). A previous work by our team demonstrated that this technology can be successfully applied to silk proteins [37].

This study addresses these challenges by systematically optimizing SS concentration methods, including evaporation, rotary evaporation, dialysis, and a novel cryo-lyophilization approach. The cryo-lyophilization method uniquely overcomes the challenges of maintaining SS’s amorphous structure, avoiding the β-sheet conformations often induced by traditional lyophilization. This ensures water solubility and preserves gelling properties, which are critical for biomedical applications. Furthermore, the integration of scCO_2_ sterilization provides a novel, mild alternative that effectively inactivates microbial contaminants without compromising SS’s bioactivity or functional properties. By combining these innovations, we aim to produce an off-the-shelf SS powder suitable for diverse biomedical applications.

## 2. Results and Discussion

### 2.1. Preliminary Optimization

#### 2.1.1. Rotary Evaporation Concentration

The literature lacks comprehensive information regarding the conditions necessary for extracting SS using rotary evaporation and a thorough analysis of the effects of various parameters. Hence, a preliminary study assessed the influence of temperature, pressure, and rotation (Supplementary Material SI-1).

Regarding the temperature, it was found that temperatures below 60 °C are not suitable for performing protein concentration, as the longer concentration time required leads to protein denaturation. In addition, higher temperatures hamper process control. Denaturation occurs not only due to temperature but also as a result of prolonged exposure to mechanical agitation and pH shifts during the process, as reported in other studies [38]. Reduced solubility at lower temperatures can also lead to protein aggregation, further complicating concentration [39,40]. Conversely, higher temperatures (>60 °C) may accelerate evaporation, but often result in protein denaturation due to excessive heat exposure [40]. In light of these findings, 60 °C was identified as an optimal temperature, balancing efficiency and preservation of sericin’s functional properties, and was set to conduct further tests.

An initial experiment was performed on a rotavapor connected to a vacuum pump that did not allow for pressure control. It was found that the losses of SS into the receiving flask were too high, and an experimental set-up with pressure control was implemented. Low pressures (250–500 mbar) can be used efficiently to concentrate SS. Experiments without a vacuum were also performed; however, after a few hours, the SS started to degrade. Regarding the rotary flask, increasing rotation from 120 rpm to 240 rpm did not cause any significant difference. To compare with the other concentration techniques, SS solutions in the rotavapor were obtained using 60 °C, 120 rpm, and 500 mbar (2.5 wt. %).

#### 2.1.2. Concentration Calibration

SS spectra (Supplementary Material SI-2) show that increasing SS concentration leads to an increase in the peak’s amplitude at 275 nm, attributed to the absorption of amino acids.

The concentration of the protein was determined from a calibration curve obtained from the absorbance (recorded at 275 nm) of a series of SS solutions in distilled water with a concentration range from 5 mg/mL to 30 mg/mL (Abs = 3.48Conc − 11.6; R^2^ = 0.961). Absorption spectra were obtained with a UV–visible NanodropOne^®^ spectrophotometer (Thermo Fisher Scientific, Madison, WI, USA). Each sample was analyzed in triplicate and the results given as mean ± SD. This consists of the first step to optimize and standardize SS.

### 2.2. Physicochemical Properties

#### 2.2.1. SS Extraction and Secondary Structure (CD, Cryo-SEM)

SS was extracted using boiling water, which is reported to allow the preservation of the protein’s intrinsic properties [19]. Although reagent-free approaches are increasingly being selected for tissue engineering, high-temperature and high-pressure (HTHP) autoclaving is often the selected method. This is due to the higher extraction yields compared to boiling water. According to the literature, HTHP yields can reach 80% [41], while the implemented process resulted in a yield of 27–32%.

Despite the extraction’s productivity being a disadvantage, there are other aspects to consider, such as the molecular weight (Mw) and secondary structure that affect the gelling process as well as the physical properties [27]. As reported in a previous study, the molecular weight of SS after extraction in boiling water ranges from 40–400 KDa, while after HTHP the Mw shows distinct bands at 25, 66, and 90 kDa and a broad band at 205 kDa [27]. This reduction in Mw with HTHP is associated with hydrolysis of the high molecular weight macromolecules, leading to smaller and broader size distributions, which in turn alters the final physicochemical properties of the material [42].

**Figure 1:**
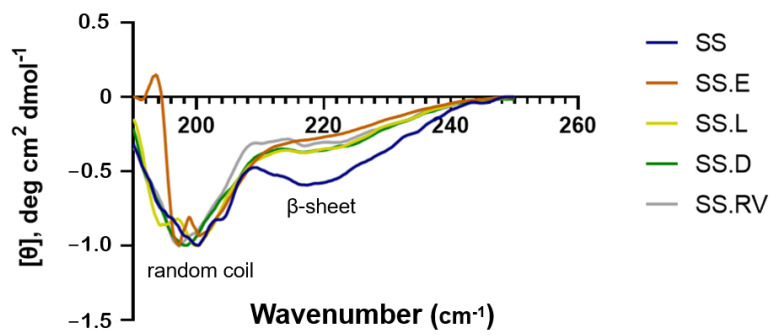
CD of SS solution after extraction in boiling water (SS) and after concentration using different methods (SS.E: evaporation, SS.RV: rotary evaporation; SS.L: cryo-lyophilization; SS.D: dialysis). The data shown represents the average of 3 readings.

Regarding the secondary structure, CD spectra of the extracted and concentrated SS solutions showed strong negative bands around 200 nm assigned to the random coil conformation and a weak negative band around 220, suggesting a β-structure (Figure 1) [43,44]. The absence of any positive bands at around ~190 nm is associated with a lack of (or residual presence of) α-helices [45]. These structures are typically found in proteins like collagen and elastin, present in connective tissue, where mechanical strength and elasticity are critical for its biological function [46]. Although SS extracted in heat lacks a significant α-helix content, its β-sheet, random coil structures, and polar functional groups offer unique properties that are highly advantageous for biomedical applications [47]. The β-sheet structures contribute to its mechanical stability and controlled degradability, while the polar functional groups enhance its hydrophilicity, biocompatibility, and ability to interact with a variety of therapeutic molecules. These structures primarily determine the material’s functional properties, such as gelation, mechanical performance, and flexibility, which are critical for applications like tissue engineering and drug delivery [47,48]. According to the literature, the obtained CD peaks are characteristic of SS after the degumming process [43]. The secondary structure fractions (%) predicted in DichroWeb corroborated the presence of these conformations (Supplementary Material SI-3). The data output was calculated through a comparison of the CD raw data (~260–190 nm) with a wide range of protein spectra datasets and their associated secondary structures, generating a reconstructed spectrum of the best-fit solution overlying the experimental spectrum [49,50]. 

The different concentration techniques that were used did not appear to cause significant changes in the secondary structure of SS, as compared with the initial solution (Figure 1). This was also valid for the cryo-lyophilization treatment (SS.L), where the water was fully removed.

During the process of water freezing, a high propensity for protein self-assembly has been described, which in turn leads to β-sheet structure formation [32]. This phenomenon has also been reported in our previous work on silk fibroin [51], in which highly organized lamellar scaffolds were produced by varying silk solution concentration and freezing parameters. For the lowest freezing temperature used (−80 °C) and highest fibroin concentrations tested (6–8 *w*/*v* %), a preferential growth direction was imposed on the ice crystals [51]. Hence, the structural properties obtained during freeze-drying are mainly regulated by exploiting the kinetics of ice formation, and fast freezing can prevent protein self-assembly, thus preserving the initial protein self-assembly.

In the work of Arango and co-authors [25], different freezing temperatures on the final structure of SS scaffolds were studied (−35 and −80 °C). The results showed that a higher percentage of crystalline structures is obtained for higher freezing temperatures: the spatial arrangement of SS froze at −35 °C, resulting in ≈9% random coil conformation and a β-sheet content of ≈37%. For lower temperatures (−80 °C), although the amorphous phase conformation of SS is better preserved (≈20%), the percentage is still lower than that of β-sheets (≈40%) [52].

Our results (Figure 1) (Supplementary Material SI-3) show that the content of β-sheets in the initial solution does not significantly increase when compared with the regenerated solution after freezing with liquid nitrogen (−196 °C) and cryo-lyophilization (SS.L). Indeed, the β-sheet content increases from 41.7 to 42.2%, which potentially indicates an increased gel permeability and reduction in mechanical constraints [49,50,53].

#### 2.2.2. Spectroscopical Characterization (XRD, ATR-FTIR, Raman, NMR)

**Figure 2:**
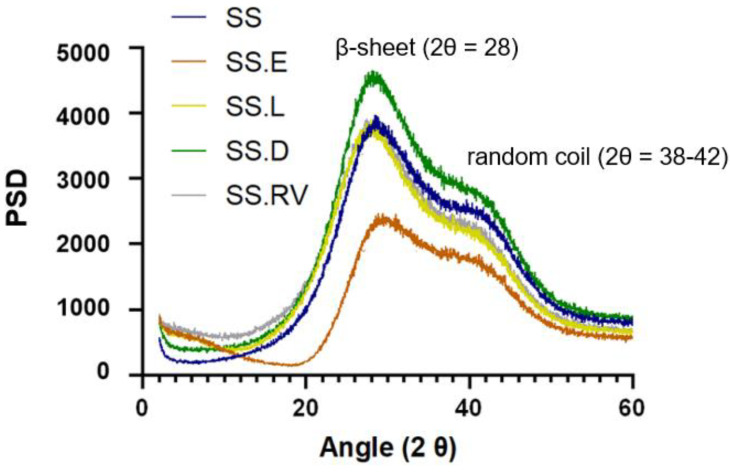
XRD spectra of the SS solutions.

The crystalline structure of the SS solutions was investigated using X-ray powder diffraction (XRD) (Figure 2).

A diffraction peak with broad intensity at 2θ = 28 was verified for all samples. In silk protein materials, this pattern is attributed to the β-sheet crystalline structure [54,55]. In silk fibroin, this peak is sharper, due to the higher percentage of crystallinity. According to the literature, adding an increasing amount of SS to a fibroin material results in a more amorphous structure [56]. In addition, a shoulder at approximately 38–42 degrees was registered, characteristic of random coil conformations and the presence of highly polar amino acids (Figure 2) [57].

Contrary to SS solutions obtained through boiling water, SS after HTHP exhibits a broad diffraction peak at 2θ ≈ 20 that indicates the conversion of the random coil structure into the β-sheet structure [58]. Differences in HTHP SS after different processing methods result in differences in crystallinity, as reported by Rocha et al. [59]. After freezing at −86 C, the resulting lyophilized powder exhibited increased crystallinity in X-ray diffractograms. As X-ray diffractogram patterns of SS after extraction in boiling water and SS.L displayed the same behavior, no conformation changes were registered, in line with the CD [59].

While the XRD spectra of SS after extraction and of SS obtained through concentration using rotary evaporation and cryo-lyophilization (SS.RV and SS.L) were identical, the intensities of the conditions attributed to dialysis and evaporation were slightly different. This takes into consideration that the gels were prepared consistently, in terms of concentration, composition, and volume. For SS.D, the presence of a more intense and well-defined peak can indicate an increase in crystallinity when compared to the other concentration methods. On the other hand, SS.E has a narrower distribution, which can indicate that the sample is more amorphous. Unlike the sample preparation for CD, in which the specimens were diluted so as not to jellify inside the analysis cell chamber, in XRD the samples were analyzed in jellified form. This can explain why certain differences in the secondary structure might not be visible.

**Figure 3:**
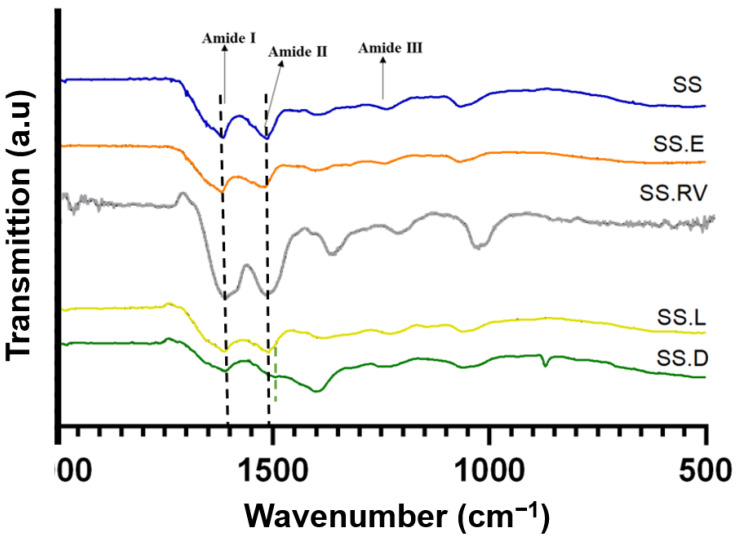
ATR-FTIR spectra of SS-based formulations with amide bands assignment. The data shown represents the average of 4 readings.

Figure 3 shows the FTIR spectra for the various SS solutions, and Supplementary Material SI-4 includes the identification of the distinct functional groups and amide I peak deconvolution.

Typical absorption bands in the SS protein, such as amide I (1600–1700 cm^−1^), II (1504–1582 cm^−1^), and III (1200–1300 cm^−1^) [8,60] were detected for all samples at around 1649 cm^−1^ (C=O stretching in carbonyl group within amidic backbone), and 1528 cm^−1^ and 1238 cm^−1^ (phenolic hydroxyl functionalities of tyrosine residues of SS) [61]. The peaks at 1395 cm^−1^ can be attributed to C=O symmetry stretching, and peaks at 1067 cm^−1^ can be attributed to C-O-C and C-O stretching vibrations [62]. The peak at 2989 cm^−1^, observed for SS after extraction and concentration using rotary evaporation (SS.RV) and dialysis (SS.D), can be attributed to the C-H stretching vibration [62].

When analyzing the difference in ratios between amide I and amide II, it was visible that this ratio changes for SS.D as a result of a shift of amide II (Supplementary SI-4). This ratio is proportional to the amount of hydrogen bonding between the carbonyl and the secondary amine structures of the peptide repeats [63], and can be associated with a change in the secondary structure [64,65].

As a result of amide I peak deconvolution, the presence of both random coil and β-sheet characteristic peaks in all SS solutions studied was verified (Supplementary Material SI-4) [66]. This was also verified by Raman spectroscopy (Supplementary Material SI-5), which evidenced the presence of β-sheet structures, in which the typical wavenumbers are 1674–1672 cm^−1^ and 1242–1227 cm^−1^ for amide I and amide III modes, respectively [67].

#### 2.2.3. Morphology and Organization

**Figure 4:**
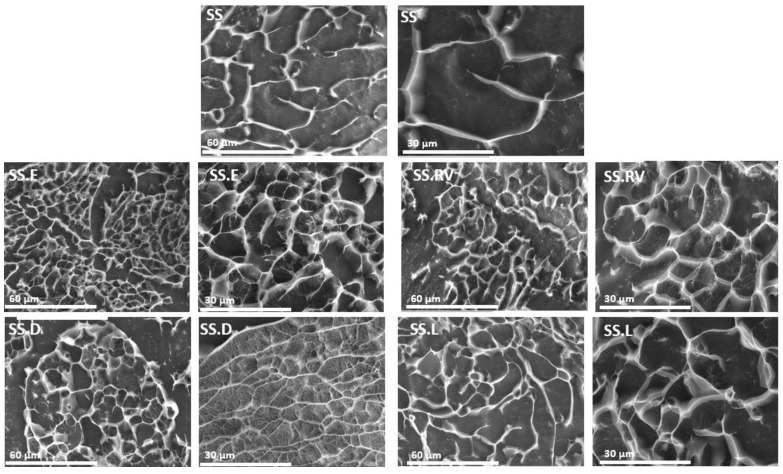
Cryo-SEM images for SS after extraction and concentration (magnification = ×1000 and ×2000).

The morphology of the SS processed under varying conditions was analyzed through Cryo-SEM (Figure 4). All protein-based hydrogel structures can generate porosity after water removal [68,69], which increases with a decrease in protein content (SS.E, SS.RV, SS.D, and SS.L). The effect of SS concentration on the final porosity was also reported in the work of Tao et al. [70], which additionally showed that lower freezing temperatures resulted in a bigger average pore size and density [70]. Contrary to silk fibroin, which results in oriented and organized structures after freeze-drying, the structure of SS for most of the tested samples did not follow a specific pattern, owing to its amorphous nature [51]. However, for the SS.D condition, portions of the sample adopted a more aligned structure (Figure 4).

Flash freezing, or instant freezing, significantly reduces the time available for molecular alignment or aggregation during ice crystal formation. Unlike traditional freezing methods, which are slower and allow for more extended interactions between protein molecules, flash freezing creates smaller and more uniformly distributed ice crystals. This rapid process minimizes protein conformational changes and aggregation, thereby preserving the secondary structure more effectively [51].

In contrast, slower freezing methods often lead to larger, less uniform ice crystals that can disrupt protein stability. As noted in the literature, the morphology of freeze-dried structures is directly influenced by the freezing rate and temperature gradient, with slower freezing rates resulting in undesirable structural variations [51].

#### 2.2.4. Rheological Behavior

**Figure 5:**
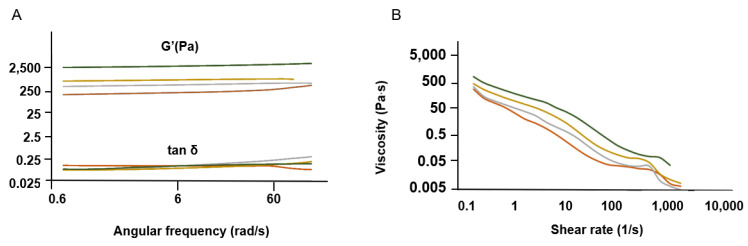
Rheological properties of SS solutions (final concentration of 1 wt. %). (**A**) Elastic modulus G′ and tan δ and (**B**) viscosity (*n* = 3).

The viscosity of the SS gels was tested to assess how the different concentration methods affected their rheological behavior (Figure 5). This is particularly relevant for SS protein due to its gelling properties [71]. SS’s self-assembling nature can be leveraged for the development of natural hydrogels [60].

The curves obtained are representative profiles of a hydrogel matrix in which tan δ < 1, which indicates that G′ > G″ (Figure 5A), consistent with a material that behaves like an elastic solid [72,73]. The SS gelation phenomenon was preserved, even at low concentrations (1 wt. %), after the different concentration methods [74]. This was an interesting finding, as HTHP extraction affects the gelling properties of SS, with a minimum concentration of 3 wt. % being required for gel-like behavior [75].

According to the frequency sweep tests, G′ is frequency-independent up to approximately 50 rad/s. In addition, the G′ of SS.D is higher, indicating the presence of a stiffer gel (less viscous flow behavior) (Figure 5A). This can be linked to a more organized structure, as exhibited by XRD (Figure 2).

The viscosity profile reveals shear thinning behavior for all SS solutions analyzed, with decreasing viscosity under shear strain (Figure 5B). This profile is expected for concentrated polymer solutions. SS after dialysis (SS.D) also exhibits higher viscosity. The longer concentration period during dialysis might have favored the molecular self-assembly of SS, which would explain the differences observed in Cryo-SEM (Figure 4).

The remaining experimental conditions (SS.E, SS.RV, SS.L) ensured the preservation of SS properties post-processing. This was anticipated for SS.E, as it involves concentrating the solution by evaporation, a method validated in previous studies [27,76]. In this work, optimizing the methodology to ensure greater control was essential for consistency and reproducibility, which is a common challenge with natural proteins [19].

For SS.RV, examining the influence of different parameters allowed the development of a viable methodology, defining the thresholds for temperature, pressure, and rotation that did not affect the final properties of SS (Supplementary Material SI-1). After SS.E and SS.RV, an additional dilution step is usually required to obtain the desired final concentration.

The freeze-drying method (SS.L) posed the highest risk of altering the SS conformation due to the freeze-drying process and subsequent dissolution [25,32]. As previously mentioned, this process can increase the crystallinity, reducing SS solubility and impairing its gelling ability [48]. However, using boiling water extraction combined with flash freezing in liquid nitrogen (−196 °C) preserved the amorphous structure of SS, resulting in a dissolvable powder. This methodology offers advantages over other optimized techniques, including improved storage, easier transportation, extended shelf life (which is only up to one week for a refrigerated SS solution), and better control over the final concentration by using the required amount of SS powder directly.

Hence, after optimizing SS concentration techniques and evaluating the resulting physicochemical properties, SS.L was selected to tackle another challenge in protein processing: SS sterilization.

### 2.3. Validation of scCO_2_ Sterilization

#### Turbidity Tests and Protein Conformation

According to previous studies, silk proteins are highly affected by the sterilization method applied [77]. Gamma irradiation, while achieving sterility at doses of 25–50 kGy, induces structural changes that accelerate scaffold degradation rates and increase material stiffness, as reported in studies on silk fibroin scaffolds [78,79]. Similarly, EtO sterilization, though less impactful on structural properties, leaves cytotoxic residues that require prolonged leaching, reducing cell proliferation by 30–40% [78]. scCO_2_ sterilization has a high potential to be adopted as a standard terminal sterilization method, not only because it works at low temperatures but also because of its minimally reactive nature and ability to diffuse into complex shapes [80,81].

**Figure 6:**
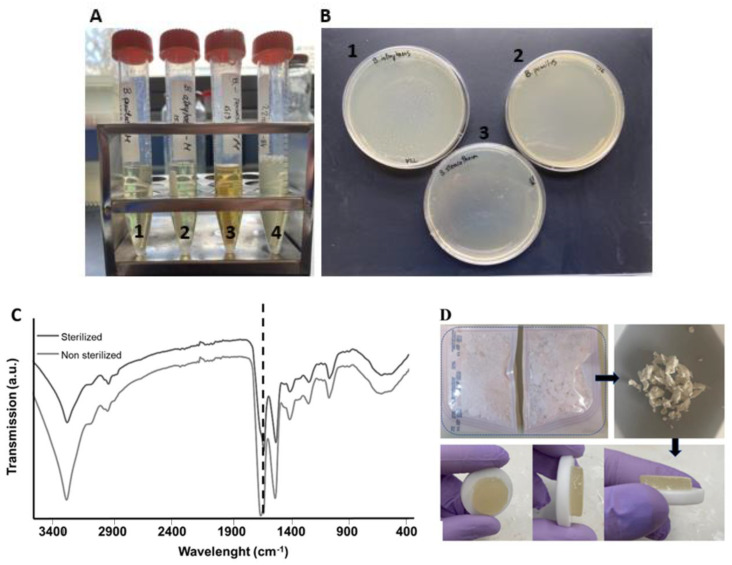
(**A**) Turbidity results after 12 days for 1: sterilized *B. pumilus*; 2: sterilized *B. atrophaeus*; 3: sterilized *B. stearothermophilus* and 4: non-sterilized *B. pumilus* (positive control); (**B**) Culture of the medium collected from the turbidity tests and incubated during 2 days in TSA plates (1: *B. atrophaeus*; 2: *B. pumilus*; 3: *B. stearothermophilus*). (**C**) ATR.FTIR spectra of lyophilized SS before and after sCO_2_ sterilization, in which the amide I peak position was maintained (dashed line). (**D**) SS raw material in sterilization pouches before sCO_2_ and after dissolution in PBS (2 wt. %).

Turbidity tests conducted after scCO_2_ sterilization showed that after 12 days of incubation, all the biological indicators were sterile (indicated by the clear solutions), as presented in Figure 6A. The color change registered in A3 can be attributed to the type of spore tested. In the positive control (A4), the medium is turbid, indicating bacteria growth. After further culture of the medium collected from the turbidity tests in TSA plates (Figure 6B: 1, 2, and 3), the results obtained were identical, with no indication of contamination for the sterilized specimens. Hence, according to these results, the sterilization method was terminal and can be used with SS.

Regarding protein conformation, it was possible to verify that scCO_2_ sterilization did not change the protein structure through analysis of the chemical structure of lyophilized SS, as there was no shift in the amide I peak (Figure 6C). This was further supported by the shape and color preservation of SS after sterilization, and its gelling properties after dissolution (Figure 6D).

### 2.4. Lyophilized vs. Commercial Sericin

Although there are a few retailers in the Asian market selling SS powder online (such as Huzhou Xintiansi Bio-tech Co.; AOTESI BIOCHEMISTRY IND Co., Ltd. HUZHOU; Xi′an Julong Bio-Tech Co.; Guangdong Kelaiya Biotechnology Co.; Wuhan Disel Biotechnoloy Co., Ltd.; Shaanxi Hizer biotech company Shaanxi Yuantai Biological Technology Co., Ltd.), information about the properties of the material, as well as its preparation and applicability, is limited.

SS.L retained its intrinsic gelling properties after dissolution of 1 wt. % in PBS, confirmed by rheological analysis (G′ > G″) (Supplementary Material SI-6A). Commercially sourced SS lost its gelling properties after dissolution using the same conditions, staying in liquid form (Supplementary Material SI-6B). From the FTIR analysis, it was possible to observe that the secondary structure is identical for all studied conditions (SS.L and commercial SS) with a clear overlap of amide I peaks (Supplementary Material SI-6C). SEM analysis further indicates that the surface of the lyophilized SS exhibits greater roughness compared to the commercial SS (Supplementary Material SI-6D), but is also equivalent to the morphology of SS after extraction in boiling water. This roughness can serve as anchor points for cell membrane receptors, promoting the formation of focal adhesions and enhancing cell adhesion, consistent with our results and other SS-related studies [27,76,82]. However, some research has suggested that the effects of surface roughness on cell–surface interactions are inconclusive or may depend on additional factors [83]. Further studies are necessary to evaluate these dependencies.

**Figure 7:**
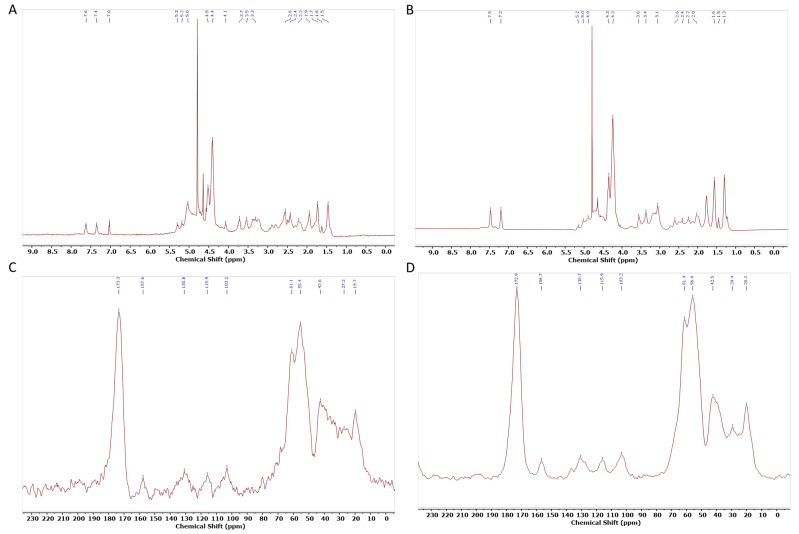
NMR analysis of (**A**); SSL ^1^H-NMR S, (**B**); ^1^H-NMR commercial SS (**C**); ^13^C CP-MAS SSL (**D**); SSL D: ^13^C CP-MAS commercial SS.

To further evaluate conformational differences, structural information was collected through NMR. The assignment of 1H-NMR peaks was based on previous assignments of silk fibroin and small peptides (Figure 7). In the spectrum, the chemical shifts of the main amino acid residues can be observed at the following ppm values: 7.6 and 7.4 ppm for Tyr residues; 4.1, 4.4, and 4.5 ppm for CαH in Gly, Ala, and Ser residues, respectively; 3.7, 3.5, and 3.3 ppm for Ser, Asp, and Ala residues, respectively; and 1.9 ppm for Arg and (1.6 and 1.5) ppm for Lys. These chemical shifts closely resemble the 1H chemical shifts of random coil structures, as determined by different authors [44,84]. Similarly, in the CP/MAS spectra, the corresponding peaks for Ser Cα, Ser Cβ, and Gly Cα were observed at 173.3, 55.4, 61.1, and 42.6 ppm, respectively. This indicates that SS maintains a largely random coil structure even after cryo-lyophilization [85].

When comparing 1H-NMR SS.L with commercial SS, it is possible to observe that SS.L has an extra peak between 7 and 7.6 ppm. This could be due to the retention of aromatic residues in the SS.L sample, enhancing the interaction potential between protein molecules [86]. Additionally, the peak at 4.1 ppm, associated with α-protons of amino acids, has a higher intensity, which can suggest differences in the solubility or aggregation state of the protein [87].

According to the results, SS.L demonstrates superior gelling properties and structural integrity post-processing, providing a more consistent and reliable material for end-use applications. Unlike commercial SS, which often lacks detailed characterization and reproducibility, SS.L benefits from optimized processing methods that minimize batch-to-batch variability. These improvements translate into a more predictable production pipeline and reduced material waste, ultimately enhancing economic viability. Moreover, the cryo-lyophilization process facilitates the production of a dry, stable powder with an extended shelf life, reducing storage and transportation costs. Together, these factors underscore the potential of SS.L as a scalable and cost-effective alternative for industries ranging from biomedicine to cosmetics.

### 2.5. Biological Assessment

**Figure 8:**
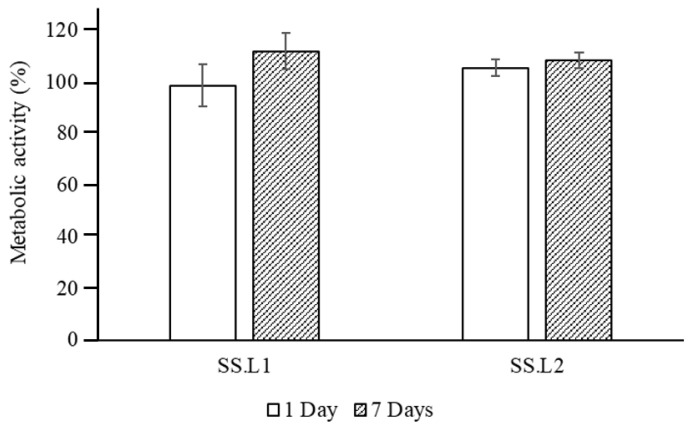
Normalized metabolic activity of HDFs cultured using extracts of SS.L1 (1 wt. %) and SS.L2 (0.5 wt. %) collected after 1 and 7 days (*n* = 8).

In vitro tests using HDFs demonstrated that sterilized SS.L is cytocompatible, with cell metabolic activity superior to 98% for extracts from 1 and 7 days (Figure 8). Furthermore, the differences between SS.L1 (1 wt. %) and SS.L2 (0.5 wt. %) are not statistically significant (*p* > 0.05), suggesting that both concentrations result in similar biological behavior.

**Figure 9:**
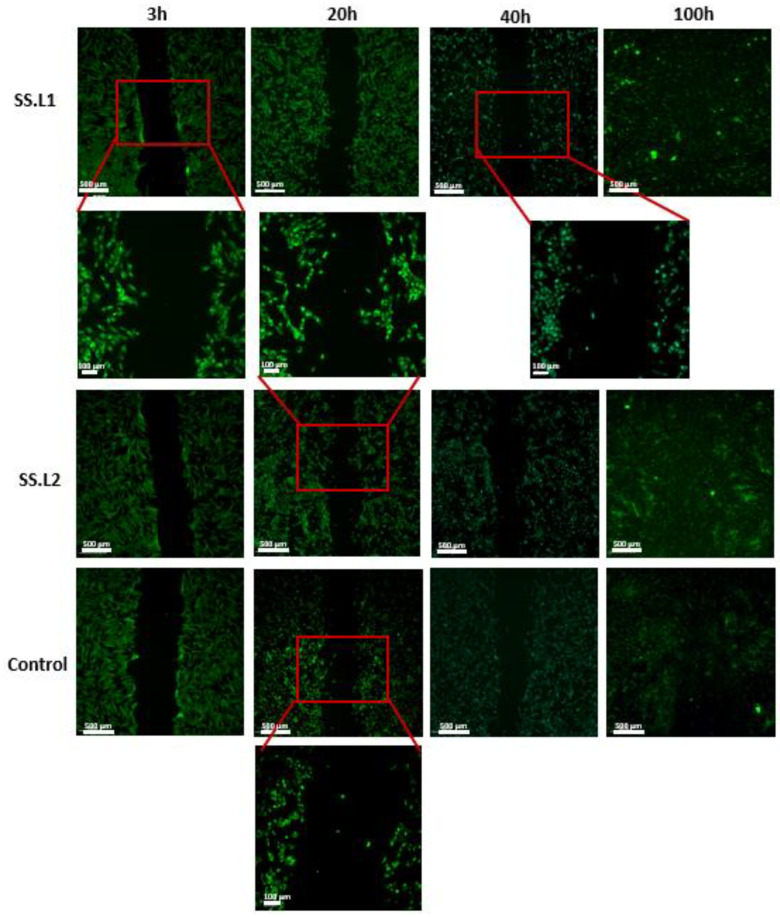
Fluorescence images collected for the wound-healing assay after 3 h, 20 h, 40 h, and 100 h for SS.L1, SSL2, and control groups (magnification 10× and 40×) (*n* = 3).

The wound-healing assay confirmed the excellent biological properties of the collected extracts, which did not hinder cell proliferation (Figure 9). Over time, the scratch simulating the wound decreased until it filled at the end of 100 h. Over this time, the migration of cells from one side of the wound to the other was evidenced. Since SS dissolves in aqueous media when it is not crosslinked [88], it was released into the culture medium without compromising cell growth. In fact, SS has been effectively used as a supplement in culture media [89]. These results are consistent with reported results in the literature concerning the biological properties of SS [90], but also demonstrate the effectiveness of the sterilization method implemented.

The findings of this study represent an important step toward continuing to integrate SS into different applications, particularly in the development of sterile, gel-like structures. This approach is especially promising in tissue engineering and regenerative medicine. Hydrogels developed from SS using the methodology employed in this study show great potential, particularly in skin regeneration. The antioxidant, anti-inflammatory, and collagen-stimulating properties of SS make it an ideal candidate for wound healing and dermal reconstruction [27,91]. Moreover, advances in hydrogel design, such as anisotropic conductivity and multi-stimulus responsiveness, as demonstrated in recent studies, could inspire the incorporation of SS into bioactive and mechanically tunable hydrogels for applications requiring enhanced flexibility, degradability, and responsiveness [92].

Another application gaining increasing attention is drug delivery systems. SS’s biocompatibility and structural adaptability make it an excellent vehicle for the controlled release of therapeutic agents. Recent studies highlight its ability to encapsulate drugs efficiently and release them in a controlled manner, particularly in pH-sensitive environments. This opens up new avenues for targeted cancer therapy and other precision medicine applications [93]. Additionally, the structural properties of SS hydrogels could be further enhanced by integrating dynamic stiffness adjustment, similar to approaches used in structural fabrics, which allow for controllable mechanical properties and robustness under varying conditions. Such developments could further expand the scope of SS-based materials in advanced biomedical applications [94].

Moreover, the use of silk proteins such as SS in biofabrication and bioprinting is also rapidly expanding. Advances in SS-based bioinks have demonstrated their potential in creating complex 3D constructs, including vascularized tissues and organoids. However, there are currently no materials where SS is the primary component with its intrinsic gelation properties preserved. This feature could enable cell encapsulation and leverage sericin’s potential as both an organic matrix and a nourishing medium for cell maintenance. The material developed in this study, with its shear-thinning properties, could serve as an exciting platform for the development of such systems [81,95].

## 3. Conclusions

For the first time, a study was carried out optimizing and comparing different concentration methods from SS solutions extracted through boiling water. The results show that concentration by dialysis might lead to the formation of a higher content of β-sheets. On the other hand, evaporation, rotary evaporation, and cryo-lyophilization allow the preservation of SS’s physicochemical properties. The methods presented allow for greater control over SS processing, crucial for reducing bath-to-batch variability.

In particular, cryo-lyophilization showed high potential to generate an off-the-shelf material. The ready-to-use powder can be easily processed and applied to develop SS-based biomaterials. Thus, scCO_2_ sterilization of this SS system was validated as an alternative to standard sterilization procedures, which use harsh processing conditions. In vitro tests using HDFs demonstrated the high cytocompatibility of the obtained lyophilized powder. Therefore, this study represents a crucial step in identifying the optimal starting point for applying SS in future applications. The developed methodology also opens new avenues for the creation of more complex downstream applications, particularly in the development of gels and crosslinked hydrogels that act as a matrix for cell growth. By preserving the intrinsic properties of sericin, this approach enables the creation of advanced systems, such as cell-encapsulated hydrogels and bioinks, expanding the potential for tissue engineering and regenerative medicine.

## 4. Materials and Methods

### 4.1. Sericin Extraction

Bombyx mori silkworm cocoons (5 g) were cut into small pieces, cleaned, and immersed in 500 mL of deionized water (1:100 *w*/*v*) at boiling temperature for 60 min. According to a previous study, the Mw ranges from 40–400 KDa [27]. The silk used was genetically controlled at the University of Padua, Italy (Research Centre of Agriculture and Environment (AA)—Sericulture Laboratory of Padua), which is a global center of sericulture innovation, quality, and traceability. The genetic control of silk production ensures consistent amino acid composition, which directly influences SS’s bioactivity and gelation properties. Cocoon breeding and manipulation are carefully performed under aseptic conditions, according to international standards at APPACDM Sericulture, Portugal, Castelo Branco. After extraction and processing in boiling water, the silk threads were manually removed, and the resulting solution was filtered to a final volume of ≈100 mL (Whatman 1113-150St. Louis, MI, USA) to remove any remaining impurities. The purified solution was then used for concentration, employing the various methods as follows: evaporation (SS.E), rotary evaporation (SS.RV), cryo-lyophilization (SS.L), or dialysis (SS.D). Obtained SS stock solutions were kept at 4 °C for further experiments.

### 4.2. Sericin Concentration Methods

#### 4.2.1. Evaporation

Evaporation to reach the desired concentration was adapted from previous works [27]. Briefly, a given solution of SS in an open beaker was heated, and process control was achieved by fixing the temperature at 100 °C and monitoring with a thermocouple. A stirring magnet was used at 400 rpm. The initial volume of the SS solution was kept at 20 mL and a final volume of ≈5 mL of concentrated SS was obtained.

#### 4.2.2. Rotary Evaporation

SS was also concentrated in a rotary evaporator (BUCHI Rotavapor™ RII, Sant Just Desvern, Spain). Initially, the influence of the temperature (40, 50, 60, 90 °C), rotation (120, 240 rpm), and pressure (250, 500 mbar) (Vacuum controller CVC 3000) was studied. Thereafter, the experimental condition which led to a higher SS concentration was selected to perform further studies (60 °C, 120 rpm, and 500 mbar). The initial volume of the SS solution was kept at 20 mL and a final volume of ≈1 mL of concentrated SS was obtained.

#### 4.2.3. Cryo-Lyophilization

A given SS solution was instantaneously frozen in liquid nitrogen and stored at −80 °C. Flash freezing was performed to reduce or prevent changes in the secondary conformation. Afterwards, the solution was placed in the freeze-drier under vacuum and collected after 24 h. The resulting SS powder was kept in the desiccator. Preliminary tests confirmed that complete dissolution of the material was achieved in both water and PBS. For the characterization techniques performed, the samples were rehydrated in PBS at 100 °C.

#### 4.2.4. Dialysis

The SS solution was concentrated using a method adapted from the literature [96]. Briefly, 50 mL of the SS solution after extraction was placed into a dialysis membrane (Spectra/Por™ dialysis membrane with pre-wetted RC tubing MWCO: 3.5 kD) and immersed in a 20 wt. % poly (ethylene glycol) (PEG 20 KDa, Sigma-Aldrich, St. Louis, MI, USA) PEG solution for 3 h. A heating plate at 70 °C and a magnetic stirrer were used to ensure that the SS solution remained fluid [26].

#### 4.2.5. Determination of Sericin Yield and Concentration

To standardize the determination of SS concentration and to guarantee reproductivity between experiments, the concentration of the SS solution was determined using the dry weight methodat 105 °C for 24 h (Equation (1)).

(1) $$Dry\;weight\left(\%\right)=\frac{w_{d}-w_{c}}{W_{w}-W_{c}}\times 100$$

where *W_d_* is the dry weight of the sample with the container; *W_w_* is the wet weight of the sample with the container, and *W_c_* is the weight of the container.

SS absorbance at 275 nm was used to establish a linear relationship with its concentration (R = 0.98) [96,97,98] using a Nanodrop (Thermo Scientific NanoDrop One, (Thermo Fisher Scientific, Madison, WI, USA)).

After extraction using boiling water, the yield was calculated (Equation (2)).

(2) $$Yield\;\left(\%\right)=\frac{Final\;SS\;concentration}{Theoretical\;SS\;content}\times 100$$

#### 4.2.6. Supercritical Sterilization (scCO_2_)

**Figure 10:**
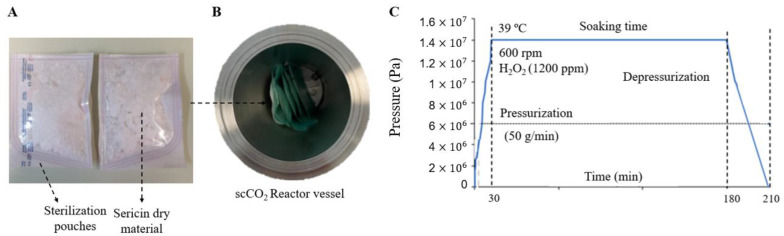
(**A**) SS dry material after processing, (**B**) reactor vessel, and (**C**) pressure–time cycle used for the sterilization process.

To evaluate the possibility of creating an off-the-shelf SS dry powder for further regeneration and use, lyophilized SS samples were sealed in sterilization pouches (3M™ Steri-Dual™) and placed inside a 2L stainless steel autoclave (Eurotechnica GmbH, Bargteheide, Germany) equipped with an agitation system (600 rpm), including 1200 ppm of H_2_O_2_ (Figure 10). The reactor was heated to 39 °C and pressurized until 140 bar at a constant pressurization rate of 50 g/min. The system was maintained in batch mode for 2.5 h. Finally, the reactor was manually depressurized until atmospheric pressure, which took 30 min.

### 4.3. Physicochemical Characterization

#### 4.3.1. Circular Dichroism

The secondary structure of the SS was evaluated using circular dichroism (CD). Triplicates of each sample were analyzed. This technique was conducted using a Jasco J-815 spectrometer (J-815, 150S, with Spectra Manager 2.0, Easton, MA, USA) equipped with a quartz cell having a pathlength of 2 mm at room temperature (25 °C). Measurements were conducted between 190 and 250 nm at a scanning speed of 50 nm/min, using a continuous scanning mode and 0.1 nm data pitch. SS solutions (SS, SS.E, SS.RV, SS.L, and SS.D) were diluted with deionized water to a final concentration of approximately 0.4 wt. %. This dilution was carried out immediately after extraction or concentration to precede the formation of a gelled material. To reduce noise, the solutions were filtered before analysis (0.2 µm) and the cuvette was cleaned between samples with detergent, methanol, and water [99]. After normalization, the spectra obtained were analyzed with DichroWeb. This software is based on the singular value decomposition method in which the data are reduced to a small number of linearly dependent vectors that form the curves common to all the data. The algorithm interacts through linear combinations of the basis curve until it converges to a curve that best fits the spectra [50,53].

#### 4.3.2. X-Ray Diffraction

X-ray diffraction (XRD) of the different SS solutions was conducted to analyze the crystalline structure. An aluminum mold was filled with 1 mL of solution (1 wt. % SS). Analyses were performed on a Bruker D8 Advance instrument with CuKα radiation (nλ = 1.542 Å) at a 0.02 step size and 0.5 s per step.

#### 4.3.3. Fourier Transform Infrared Spectroscopy

The functional groups of the different SS solutions after drying at room temperature (25 °C) were visualized using attenuated total reflectance Fourier transform infrared spectroscopy (ATR-FTIR) analysis (PerkinElmer Spectrum Two spectrophotometer, Perkin Elmer, Milano, Italy) with an attenuated total reflection attachment. All spectra were acquired using 4 accumulations and a 2 cm^−1^ resolution in the region of 4000−400 cm^−1^. Immediately before the acquisition, a background spectrum was collected with the same operational parameters. In addition, baseline point adjustment and spectra normalization were performed. Peak deconvolution was achieved by using OriginPro 2022 (version 9.9) (Method: 2nd derivate with smooth derivate method quadratic Savitzky–Golay). ATR-FTIR was also conducted after scCO_2_ sterilization to evaluate changes in the protein conformation, indicated by a shift in the amide I group. Furthermore, dissolution in PBS and the gelling properties of the protein were tested before and after submission to sCO_2_, to corroborate this evaluation.

#### 4.3.4. Raman Spectroscopy

The chemical structure of the SS gels (1 wt. %) was further evaluated using Raman spectroscopy. The Raman spectrum was measured using a Renishaw InVia Reflex Raman system (Renishaw, New Mills, Gloucestershire, UK). Raman scattering was excited using a diode laser at a wavelength of 785 nm. The laser beam was focused on the sample with a 0.85 × 100 microscope objective coupled to the system. The exposure time and the number of accumulations for the Raman measurements corresponded to 10 s and 10, respectively.

#### 4.3.5. Nuclear Magnetic Resonance

The chemical structures of SS.L and commercial SS were further evaluated using nuclear magnetic resonance (NMR). ^1^H MNR measurements were performed on a JEOL JNM-ECZ400R (Jeol LTD, Peabody, MA, USA operating at 400 MHz at 45 °C in pre-saturated conditions. SS.L was dissolved at a concentration of 2 wt. % in deuterium oxide (D2O, Merck KGaA, Darmstadt, Germany). 13C nuclear magnetic resonance (CP/MAS 13C NMR) measurement was conducted on a Bruker Avance TM400WB (Bruker Optik GmbH, Ettlingen, Germany) equipped with a wide-mouth superconducting magnet (89 mm) operating at 9.4 T. (Larmor frequenc 100.61 MHz). Samples were milled after immersion in liquid nitrogen and contained in a cylindrical ceramic rotor. ^13^C NMR spectra were acquired at ambient temperature and a spinning speed of 7 kHz, 3.50 ms width, 90 pulses, and 3 s repetition time, and a cross-polarization contact time of 1 ms. Spectra were accumulated at a frequency of 2000 scans and referenced to adamantane. Spectral analyses were performed using MestreNova 15 Software (Version 15.0.0.34764).

#### 4.3.6. Cryo-Scanning Electron Microscopy

SEM/EDS examination was performed using a high-resolution scanning electron microscope with X-ray microanalysis and CryoSEM experimental facilities (JEOL JSM 6301F/Oxford INCA Energy 350/Gatan Alto 2500). The specimens were rapidly cooled by plunging into sub-cooled nitrogen–slush nitrogen and transferred under vacuum to the cold stage of the preparation chamber. Afterwards, the different SS samples in duplicate were fractured, sublimated (‘etched’) for 300 sec at −90 °C, and coated with Au/Pd by sputtering for 50 s. The samples were transferred into the SEM chamber and studied at −150 °C.

#### 4.3.7. Viscosity Measurements

Rheology experiments were performed using triplicates of SS at a concentration of 1 wt. % and a controlled temperature of 37 °C using a rheometer (ARG2, TA Instruments, New Castle, DE, USA) equipped with a cone-plate cross-geometry (20-mm diameter).

Storage modulus (G′) and loss modulus (G″) were recorded at a frequency of 0.1–1000 Hz and 1% strain. Complex viscosity (also represented as η*) is a measure of the total resistance to flow as a function of angular frequency (ω) and is given by the quotient of the maximum stress amplitude and maximum strain rate amplitude. Viscosities were also determined by rotational shear measurements at an increasing shear rate from 0.1 to 10,000 s^−1^. Three samples were tested for each condition.

#### 4.3.8. Turbidity Tests

Turbidity tests were performed to assess the sterility of the process and f the SS powder, before and after sterilization. Commercial spore strips with 10^6^ spores of three different biological indicators, including *Bacillus stearothermophilus* (*B. stearothermophilus* ATCC 7953, Valencia, Spain) and *Bacillus pumilus* (*B. pumilus* ATCC 27,142 Valencia, Spain) were purchased from Sigma-Aldrich (Madrid, Spain). *Bacillus atrophaeus* (*B. atrophaeus* cell line ATCC 9372, Catalog number: 0953) spores were obtained from Crosstex International (Rush, NY, USA). The treated spore strips were incubated in 6 mL of TSB liquid medium at their optimal growth temperatures (37 °C, *B. pumilus* and *B. atrophaeus*; 60 °C, *B. stearothermophilus*) and bacterial growth was visually evaluated after 7 and 14 days. In addition, after 7 and 14 days of incubation, 1 mL of the culture medium was seeded in TSA plates and incubated for 1 day to determine the presence of viable microorganisms.

#### 4.3.9. Produced Sericin vs. Commercial Sericin

SS.L powder obtained by cryo-lyophilization (SS.L) and sterilized by scCO_2_ was compared with a commercial SS powder (Sigma-Aldrich Merck, S5201, Darmstadt, Germany) to evaluate differences in the gelling properties and discuss the commercial value of the developed methodology.

#### 4.3.10. Biological Behavior

Cell culture assays were performed using human dermal fibroblasts (HDFs) (Innoprot, P10856). For the in vitro biological evaluation, cells were cultured in a basal medium consisting of Dulbecco’s modified Eagle’s medium (DMEM)—low glucose enriched with 110 mg/L of sodium bicarbonate and supplemented with 20% *v*:*v* of fetal bovine serum (FBS, Gibco Thermo Fisher, São Paulo, Brasil), 200 mM L-glutamine, 100 units/mL penicillin, and 100 mg/mL streptomycin. Cells were maintained under a humidified 5% CO_2_ atmosphere at 37 °C.

#### 4.3.11. Cytotoxicity and Cell Proliferation Assays

Cytotoxicity was evaluated by measuring the cellular metabolic activity using the Alamar Blue (AB, BIO-RAD BUF012B) bioassay. HDFs were seeded in 96-well plates (Fisher Scientific, Rochester, NY, USA) at a density of 1 × 10^5^ cells/well in 100 mL of basal medium and incubated for 24 h. Afterward, media were removed and replaced by 100 mL of basal medium containing extracts from SS at different concentrations (SS.L1: 1 wt. % and SS.L2: 0.5 wt. %), collected after 24 h and 7 days. After 24 h of incubation, media were removed, 100 mL of the AB solution prepared in warm DMEM-low glucose phenol red-free (10% *v*:*v*) (Gibco) was added to each well, and the plates were incubated at 37 °C for 3 h. After this period, fluorescence was measured at 590 nm after excitation at 560 nm using a microplate reader (Synergy HT). Relative cellular viability was calculated after subtracting the fluorescence of the blank (AB solution in an empty well) from the fluorescence of the samples and normalizing it to the data of untreated cultures, which were given an arbitrary value of 100.

Data were analyzed as mean ± standard deviation, with *n* = 6 for each condition. GraphPad Software (version 8.0) was used to perform statistical analysis by implementing the 2-way ANOVA test, followed by Turkey’s method as a multiple comparison post-test (significance level was * *p* < 0.05, ** *p* < 0.01).

#### 4.3.12. Scratch Assay

SS.L1 and SS.L2 24 h extracts (*n* = 3) were used to conduct the wound-healing assay in 24-well plates with a high level of confluence (90%), seeded with 500 µL of cell suspension at 1.5 × 10^5^ cells/mL After removing the medium and washing with PBS, a marker was used to draw a line across all wells to serve as a reference, in order to assure that the pictures of the wounds were performed on the same side. Afterwards, a scratch was added perpendicular to the black line, using a 200 µL pipette tip. After dyeing (CellTrace Calcein Green, AM; Invitrogen, Bleiswijk, Netherlands), the HDFs were incubated with the extracts of the SS.L gels at 37 °C and 5% CO_2_. The scratches were documented under a fluorescence microscope after pre-determined time intervals (3 h, 20 h, 40 h, and 100 h).

## Data Availability

The data presented in this study are available on request from the corresponding authors.

## Supplementary Materials

The following supporting information can be downloaded at: https://www.mdpi.com/article/10.3390/gels11020114/s1, SI-1: Preliminary study conducted in the Rotavapor; SI-2: UV spectroscopy; SI-3: Secondary structure fractions (%) of the different SS solutions using DichroWeb; SI-4: Peak identification from the FTIR spectra and Amide I Peak deconvolution; SI-5: Raman spectra for the SS-solutions studied and peak identification; SI-6: Tests conducted with lyophilized SS (SS.L) and commercially available SS (Sigma-Aldrich, St. Louis, MI, USA).
