# Supplementary material for: Innovative Processing and Sterilization Techniques to Unlock the Potential of Silk Sericin for Biomedical Applications
Source: Gels. 2025 Feb 6;11(2):114. doi: 10.3390/gels11020114 (PMC11854797; doi:10.3390/gels11020114)
Supplement: Supplementary file 1 [file gels-11-00114-s001.zip › gels-3437596-supplementary.pdf]

Supplementary information

SI-1: Preliminary study conducted in the Rotavapor

While the conditions required to perform the extraction and optimize the techniques used to concentrate SS (SS.E, SS.L, SS.D), are reported, for SS.RV this information is lacking. Hence, a preliminary study was conducted to quickly assess the influence of temperature, pressure, and rotation (**Table SI-1**). Regarding the temperature, it was found that temperatures below 60 ºC are not suitable to perform protein concentration. In addition, higher temperatures do not lead to increased protein concentration. Thus, 60 ºC was set to conduct further tests.

An initial experiment was performed on a rotavapor connected to a vacuum pump that did not allow pressure control. It was found that the losses of SS into the receiving flask were too high, and an experimental set-up with pressure control was implemented. Low pressures, (250 – 500 mbar) can be used efficiently to concentrate SS. Experiments without a vacuum were also performed, however, after a few hours, the SS started to degrade. Regarding the rotary flask, increasing rotation from 120 rpm to 240 rpm did not cause any significant difference. To compare with the other concentration techniques, SS solutions in the rotavapor were obtained using 60 ºC, 120 rpm, and 500 mbar (2.5 wt%).

Table SI-9.1. Experimental conditions were tested in Rotavapor, and the final concentration was determined by UV-Vis.

Temperature (º C)	Pressure (mbar)	Rotation (rpm)	Final concentration (% w/v)
40			1.6
50			1.4
60	500 mbar	120 rpm	2.5
90			2.3
60		240 rpm	2.3
60	250 mbar	120 rpm	2.1

SI-2: UV spectroscopy

SS spectra (**Figure SI-2**) show that increasing SS concentration leads to an increase in the peak's amplitude at 275 nm, attributed to the absorption of amino acids.

The concentration of the protein was determined from a calibration curve obtained from the absorbance recorded at 275 nm of a series of SS solutions in distilled water with a concentration range from 5 mg/mL to 30 mg/mL (Abs = 3.48Conc - 11.6; R^2 = 0.961). Absorption spectra were obtained with

a UV/visible NanodropOne® spectrophotometer. Each sample was analyzed in triplicate and the results given as mean ± SD. This consists of the first step to optimize and standardize SS.

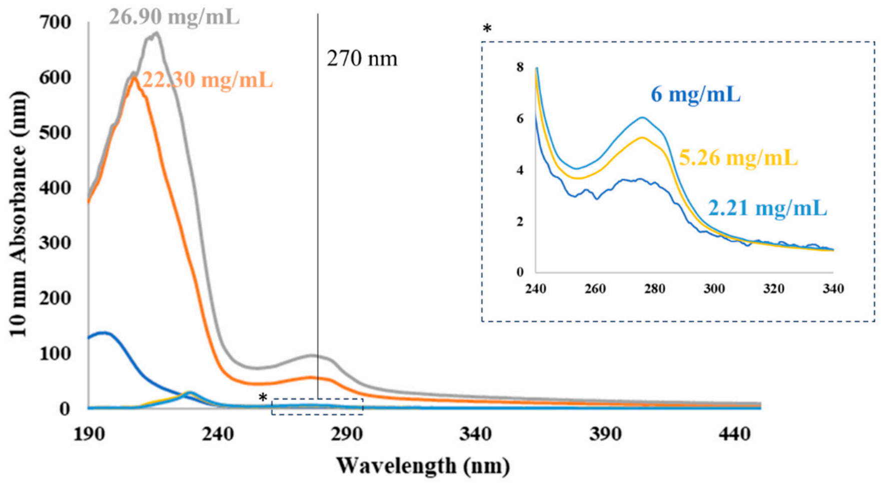

Figure SI-2. A) UV spectra of SS extracted.

SI-3: Secondary structure fractions (%) of the different SS solutions using DichroWeb

	Sericin solution				
	**SS**	**SS.E**	**SS.RV**	**SS.L**	**SS.D**
**α-Helix (%)**	5.2	4.9	4.7	5.0	4.8
**β-sheet (%)**	41.7	40.2	42.2	42.2	42.2
**Turn*1 (%)**	11.9	12.7	11.9	11.6	11.7
**Random coil (%)**	41.3	42.1	41.3	41.2	41.3
**NRMSD*2**	0.151	0.329	0.148	0.152	0.104

*1 Turn: combination of beta turns, bends and bridges.
*2 NRMS: normalized standard deviation ("goodness-of-fit parameter": indicates the correspondence between the experimental input spectrum and the back-calculated best-fit spectrum derived from the analysis).

SI-4: Peak identification from the FTIR spectra and Amide I Peak deconvolution.

Functional Group	Amide I	Amide II	Amide I/II Intensity ratios	Amide III	C=O stretching in carbonyl group within the amidic backbone	C-O-C and C-O stretching vibrations	Aggregate strands/ sidechains (Tyr, Arg, His)	Random coil	β-sheet

SS	1615	1510	1.00	1224	1391	1064	1615.5	1657	1696
SS.E	1612	1511	0.96	1231	1394	1061	1615	1664, 1660	1674, 1695
SS.RV	1610	1510	1.06	1238	1395	1068	1615	1660	1647 1693
SS.L	1613	1515	1.03	1219	1379	1064	1616	1664	1642 1700
SS.D	1610	1493	1.69	1224	1392	1064	1615	1665	1642 1669 1701

SI-5: Raman spectra for the SS-solutions studied and peak identification.

Functional Group	Amide I (1640 to 1680 cm−1)	Amide II (1500 to 1600 cm−1 interval)	Amide III – (1230 to 1310 cm−1)
SS	1653	1460	1301
SS.E	1666	1435	1230
SS.RV	1671	-	1306
SS.L	1669	1451	1239
SS.D	1668	1456	1231

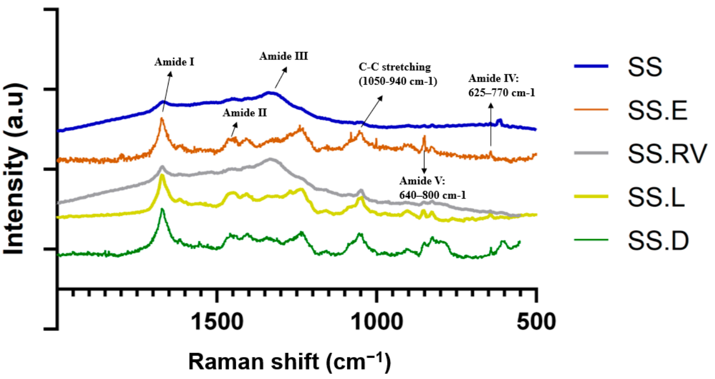

SI-6: Tests conducted with lyophilized SS (SS.L) and commercially available SS (Sigma-Aldrich)

SS powder obtained by cryo-lyophilization (SS.L) and sterilized by $scCO_2$ was compared with a commercial SS powder. Although there are a few retailers in the Asian market selling SS powder online (such as Huzhou Xintiansi Bio-tech Co.; AOTESI BIOCHEMISTRY IND Co Ltd HUZHOU; Xi′an Julong Bio-Tech Co.,; Guangdong Kelaiya Biotechnology Co.; Wuhan Disel Biotechnoloy Co., Ltd; Shaanxi Hizer biotech company Shaanxi Yuantai Biological Technology Co., Ltd.), information about the properties of the material, as well as its preparation and applicability is limited. Thus, SS powder was acquired in Sigma-Aldrich (Merck, S5201).

SS.L retained its intrinsic gelling properties after dissolution of 1 wt% in PBS, confirmed by rheologic analysis (G’>G’’) (**Figure SI-4 A**), as opposed to the commercial SS that lost its gelling properties after dissolution using the same conditions, staying in liquid form (**Figure SI-4 B**). From the FTIR analysis, it was possible to observe that the secondary structure is identical for all studied conditions (SS.L and

commercial SS) with a clear overlap of Amide I peaks (**Figure SI-4 C**). The SEM analysis further indicates that the surface of the lyophilized SS exhibits greater roughness compared to the commercial SS (**Figure SI-4 D**) but is also equivalent to SS morphology after extraction in boiling water. This roughness can serve as anchor points for cell membrane receptors and promote the formation of focal adhesions [1-3], leading to enhanced cell adhesion which is in line with the obtained results and other SS-related works.

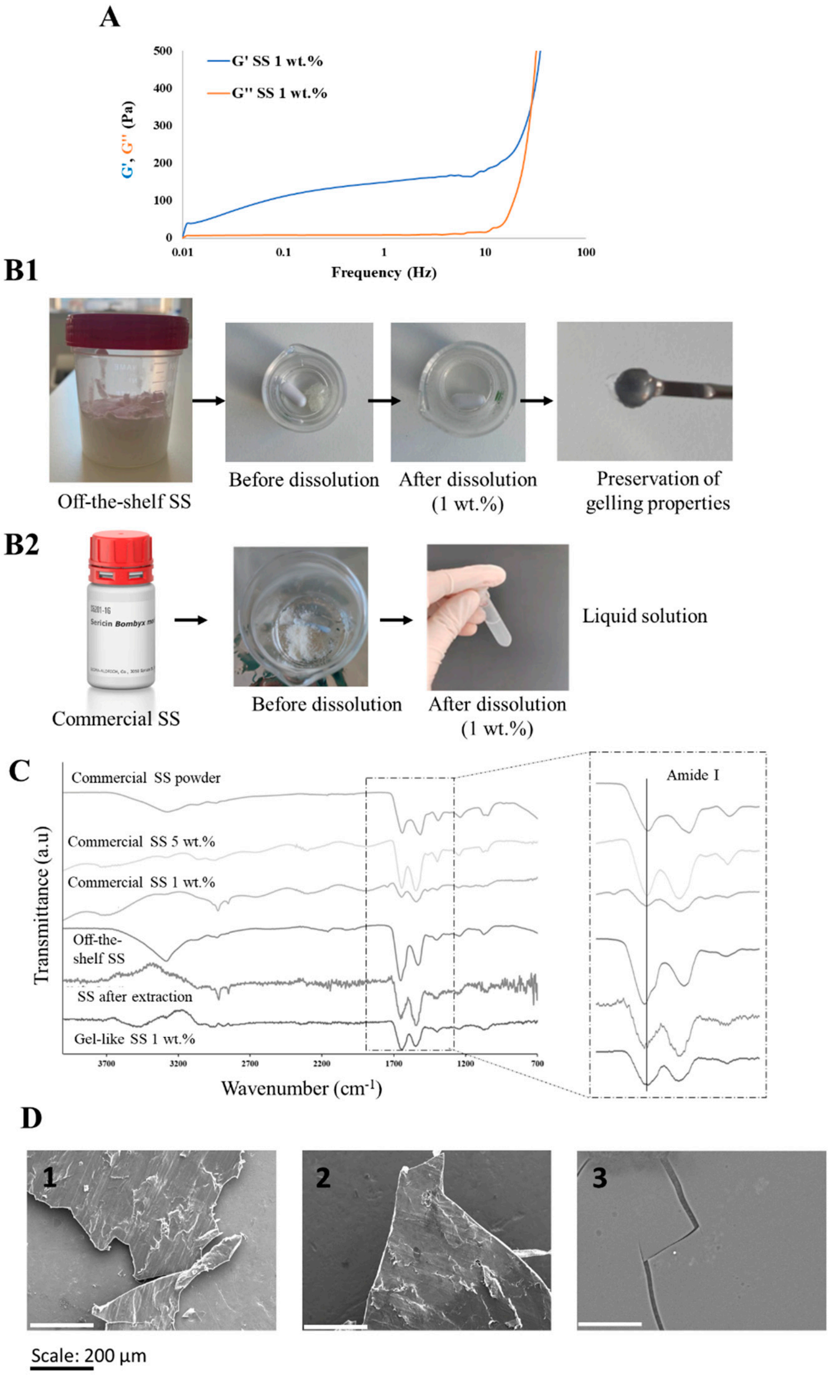

Figure SI-4: A) rheology analysis of a solution of 1wt% of SS obtained by cryo-lyophilization, at a controlled temperature of 37 ºC and with a cone-plate cross-geometry (20-mm diameter); **B)** dissolution of A- SS.L (1 wt%) and 2—SS commercial (1 wt%) and respective intrinsic gelling properties; **C)** FTIR analysis performed in commercial SS and SS obtained by cryo-lyophilization; **D)** SEM analysis performed in SS after extraction (1), dissolution of SS.L (2) and commercial SS (3).

[1] L. Cui, Y. Yao, and E. K. F. Yim, "The effects of surface topography modification on hydrogel properties," APL Bioeng, vol. 5, no. 3, Sep. 2021, doi: 10.1063/5.0046076.

[2] S. Baptista-Silva et al., "Exploring Silk Sericin for Diabetic Wounds: An In Situ-Forming Hydrogel to Protect against Oxidative Stress and Improve Tissue Healing and Regeneration," Biomolecules, vol. 12, no. 6, p. 801, Jun. 2022, doi: 10.3390/biom12060801.

[3] S. Baptista-Silva et al., "In Situ Forming Silk Sericin-Based Hydrogel: A Novel Wound Healing Biomaterial," ACS Biomater Sci Eng, vol. 7, no. 4, pp. 1573–1586, Apr. 2021, doi: 10.1021/acsbiomaterials.0c01745.
